# Effect of Self-transcendence, Self-distancing, and Family Functionality on Self-care Agency in Older Adults

**DOI:** 10.17533/udea.iee.v42n2e08

**Published:** 2024-07-05

**Authors:** Josué Medina-Fernández, Claudia Nelly Orozco-González, Nissa Yaing Torres-Soto, Diana Cortes-Montelongo, Antonio Yam-Sosa, Isaí Medina-Fernández

**Affiliations:** 1 Nurse, PhD. Professor. Email: josue.medina@uqroo.edu.mx Universidad de Quintana Roo Mexico; 2 Nutriologist, PhD. Professor. Email: oogc870223gl4@unicla.edu.mx Universidad Autónoma del Estado de México Mexico; 3 Psychologist, PhD. Professor. Email: nissa.torres@uqroo.edu.mx Universidad de Quintana Roo Mexico; 4 Nurse, PhD. Professor. Email: dicortesm@uadec.edu.mx Universidad Autónoma de Coahuila Mexico; 5 Nurse, PhD. Professor. Email: antonio.yam@correo.uady.mx Universidad Autónoma de Yucatán Mexico; 6 Nurse, PhD. Professor. Email: isai-medina@uadec.edu.mx. Corresponding author Universidad Autónoma de Coahuila Mexico; 7 Universidad Autónoma del Estado de Quintana Roo, Quintana Roo; México Universidad de Quintana Roo Mexico; 8 Universidad Autónoma del Estado de México, Estado de México; México Universidad Autónoma del Estado de México Mexico; 9 Universidad Autónoma de Coahuila, Coahuila; México Universidad Autónoma de Coahuila Mexico; 10 Universidad Autónoma de Yucatán, Yucatán; México Universidad Autónoma de Yucatán Mexico

**Keywords:** aged, adult health, family relations, selfcare., anciano, salud del adulto, relaciones familiares, autocuidado, idoso, saúde do adulto, relações familiares, autocuidado

## Abstract

**Objective.:**

To determine the effect of self-distancing, self-transcendence, and family functioning on self-care agency in Mexican older adults**.**

**Methods.:**

Correlational-explanatory design, with a sample of 253 elderly, collecting data through a simple random sampling. A personal data questionnaire was applied, the scale of: self-transcendence, the self-distancing subscale, the family APGAR and the ability to self-care in Mexican population from different demographic groups. Descriptive and inferential statistics were applied (Mann-Whitney U and a structural equation model) and the study was approved by a registered ethics committee.

**Results.:**

The study had participation from 253 elderly, with a mean age of 68.02 years, with prevalence of the female sex (60.1%); the level of education was primary school or lower (51.4%). It was observed that the group of chronic diseases had lower self-distancing (*U* = 4.449.5, *p* = 0.038) and greater self-transcendence (*U* = 4177.0, *p* = 0.008), and selfcare (*U* = 4365.5, *p* = 0.024) than the group without chronic diseases. It was also found that self-transcendence, self-distancing, and family functionality produce a positive effect of 37% on selfcare.

**Conclusion.:**

Self-distancing, self-transcendence, and family functionality explain an important proportion of selfcare in the elderly. Said knowledge permits understanding the care behavior of the elderly and, thus, propose future educational interventions by nursing to prevent or avoid functional, cognitive loss and social effects.

## Resumen

**Objetivo.:**

Determinar el efecto del autodistanciamiento, autotrascendencia y funcionamiento familiar sobre la agencia de autocuidado en adultos mayores mexicanos**.**

**Métodos.:**

Diseño correlacional-explicativo, con una muestra de 253 adultos mayores, recolectado mediante un muestreo aleatorio simple. Se aplicó un cuestionario de datos personales, la escala de: autotrascendencia, la subescala de autodistanciamiento, el APGAR familiar y la capacidad de autocuidado en población mexicana de diferentes grupos demográficos. Se aplicó estadística descriptiva e inferencial (U de Mann-Whitney y un modelo de ecuación estructural) y contó con la aprobación de un comité de ética registrado.

**Resultados.:**

Participaron 253 adultos mayores, con una media de 68.02 años, predominó el sexo femenino (60.1 %) y el grado de estudio de primaria o menor (51.4 %). Se observó que el grupo de enfermedades crónicas tuvo menor autodistanciamiento (*U* = 4.449,5, *p* = 0.038) y mayor autotrascendencia (*U* = 4.177,0, *p* = 0,008) y autocuidado (*U* = 4.365,5, *p* = 0.024) que el grupo sin enfermedades crónicas. También se encontró que la autotrascendencia, autodistanciamiento y funcionalidad familiar producen un efecto positivo de un 37 % sobre el autocuidado.

**Conclusión.:**

El autodistanciamiento, autotrascendencia y la funcionalidad familiar explican una importante proporción del autocuidado en los adultos mayores. Dicho conocimiento permite entender la conducta del cuidado del adulto mayor y de esta manera proponer a futuro intervenciones educativas por enfermería con miras a prevenir o evitar la pérdida funcional, cognitiva y afectaciones sociales.

## Resumo

**Objetivo.:**

Determinar o efeito do autodistanciamento, da autotranscendência e do funcionamento familiar na capacidade de autocuidado em idosos mexicanos.

**Métodos.:**

Desenho correlacional-explicativo, com amostra de 253 idosos, coletada por meio de amostragem aleatória simples. Aplicou-se um questionário de dados pessoais, a escala de autotranscendência, a subescala de autodistanciamento, o APGAR familiar e a capacidade de autocuidado na população mexicana de diferentes grupos demográficos. Estatísticas descritivas e inferenciais (U de Mann-Whitney e modelo de equações estruturais) foram aplicadas e tiveram aprovação de um comitê de ética registrado.

**Resultados.:**

Participaram 253 idosos, com idade média de 68.02 anos, predominou o sexo feminino (60.1%) e o nível de escolaridade era fundamental ou inferior (51.4%). Observou-se que o grupo com doenças crônicas apresentou menor autodistanciamento (U=4.449.5, *p*=0.038) e maior autotranscendência (*U*=4177.0, *p*=0.008) e autocuidado (*U*=4365.5, *p*= 0.024) que o grupo sem doenças Crônicas. Verificou-se também que a autotranscendência, o autodistanciamento e a funcionalidade familiar produzem um efeito positivo de 37% no autocuidado.

**Conclusão.:**

O autodistanciamento, a autotranscendência e a funcionalidade familiar explicam uma proporção importante do autocuidado em idosos. Esse conhecimento permite compreender o comportamento de cuidar do idoso e desta forma propor futuras intervenções educativas pela enfermagem com vistas a prevenir ou evitar perdas funcionais, cognitivas e efeitos sociais.

## Introduction

The increased prevalence of chronic diseases and the progressive aging of the population is a source of concern and occupation for diverse professionals responsible for health care.^1^ The care of the elderly is often attended to from a “physical” point of view, ignoring the psychological aspects involved in self-care agency,^1^ which could increase the demand for services and care costs, mainly generated by new diagnoses or complications, with existence of factors like self-transcendence, self-distancing, and family functionality - variables that could affect selfcare.^2^^,^^3^

Among these behavioral factors, there is self-transcendence and it is understood as the human capacity to go beyond one's own self and, as a consequence, expand personal limits through a spiritual path to give meaning to life and that is linked to a connection with the self, the environment, and with the spirit of the universe, that is, it is the meaning of one's existence.^4^^-^^6^ In turn, when addressing self-distancing, it is understood as the conscious ability to distance oneself from situations that may affect oneself, being able to confront this situation and improve behavioral processes.^7^ With the foregoing, a knowledge gap exists in the field of application of the elderly in nursing, finding an approach from chronic disease in this age group, where it is mentioned that with chronic disease in the elderly, they stop caring for themselves, derived from the physical, psychological and, family changes caused by the disease,^8^ which becomes an area of opportunity for geriatric nursing upon considering the behavior as a modifying factor of self-care agency. 

Likewise, when approaching the family, it is understood that it could be a factor that may influence upon selfcare, given that the family functionality or a functional family is achieved by promoting the integral development of its members, as well as by keeping the elderly in a favorable state of health, and with such, the elderly perceive family functioning, manifesting the degree of satisfaction with the compliance of the basic parameters of family function, such as, adaptation, participation, gain or growth, affect and resources, recognizing the family as a principal support institution of the care by nursing, highlighting the need to consider them in the factors that could modify selfcare.^9^

Lastly, when speaking of the self-care agency in the elderly, mention is made of the quality, aptitude or ability as fundamental capacity that groups the individual’s basic abilities (sensation, perception, memory, and orientation), the components of power (specific capacities related with the individual’s ability to commit to selfcare), and the capabilities for self-care operations, seeking a balance among physical, psychological and social aspects.^10^Hence, it is necessary to approach in this age group the self-care behavior in the elderly with and without chronic diseases, which separately constitute an important percentage of the global disease burden and, often, occur simultaneously, which is why they should be considered jointly and above all taking into account other intrinsic factors, like self-transcendence and self-distancing. ^11^

Thus, it is noted that nursing also participates in geriatric care, given that it addresses the functional, cognitive-psychological and social field, where the family is included within it, justifying itself as part of the Nursing Interventions Classification, such as the actions denominated as aid in modifying oneself. This way, it is anticipated that self-distancing, self-transcendence and family dynamics could modify selfcare in the elderly with chronic conditions, so researching said problem could generate interventions based on the behavior, the family and improved self-care agency. Based on what was described, the following general objective was proposed: to determine the effect of self-distancing, self-transcendence, and family functioning on the self-care agency in Mexican elderly**.**

## Methods

This was a correlational-explanatory study. The study population was comprised by elderly inhabitants of Chetumal, Quintana Roo in Mexico. The sample was obtained through the G Power^®^ program, considering an effect size of 0.10, error probability of 0.05, power of 0.91 and three explanatory factors, obtaining a sample of 253 elderly. Simple random sampling was applied for finite samples. The inclusion criteria included being an adult 60 years old or more, living in the municipality of Quintana Roo, Mexico in 2023 and who live with at least one direct or indirect relative. With respect to the exclusion criteria, the study considered the elderly with some hearing or speech limitation, and anyone with a score < 9 on the Pfeiffer test, whose indicator is the presence of risk of cognitive impairment.

A questionnaire of the population’s characteristic data was applied (age, sex, perceived economic level, current illnesses, years living with the disease, whether caring for somebody, and if suffering any chronic disease). The instruments used were: (i) The Self-transcendence scale designed by Reed^12^validated in Mexican population to measure how individuals expand their limits in different forms. The instrument has 15 items with a Likert-type scale with four response options from 1 to 4. The total score varies between 15 and 60, in which high scores in the scale indicate higher self-transcendence and low scores indicate lower self-transcendence, with a Cronbach’s alpha of 0.85;^12^(ii) the Existential Scale Adapted to measure only the Self-distancing subscale, comprised by eight Likert-type items from 1 to 6, where 1 is yes, absolutely and 6 no, absolutely. It is interpreted with data of levels of: very low (6-17 points) low (18-24 points), medium (25-31 points), high (32-39 points), and very high (40-48 points). This scale has a Cronbach’s alpha of 0.99.^13^(iii) Family *APGAR*, which shows how the family members perceive globally the level of functioning of the family unit. It has Likert-type response options ranging from: almost always (2 points) to almost never (0 points). A score from 7 to 10 suggests a very functional family, while a score from 4 to 6 suggests a moderately dysfunctional family, and a score from 0 to 3 suggests a highly dysfunctional family. It has a Cronbach’s alpha of 0.77; (iv) Self-care capacity in Mexican population from different demographic groups, which is composed of 24 items with a response format of five Likert-type alternatives, where 1 (totally disagree) means the lowest value of the capacity for selfcare and 5 (totally agree) being the highest. The results are interpreted according to the score obtained in the total sum of each of the items, where a lower score means lower self-care agency and a higher score indicates higher self-care agency; this has a Cronbach’s alpha of 0.80 in the Mexican population.^14^

The research proposal was approved by the Research Ethics Committee registered with the National Bioethics Commission (CONBIOETICA), complying with the provisions of the regulations of the General Health Law regarding research, which applied article 13 of chapter 1, second title, treating with respect and protecting participant well-being, clearly explaining the objective of the study, where the human rights of the participants were protected, their autonomy, with the right to free decision, involving collection of their data, respecting confidentiality, and anonymity if desired, without seeking to cause discomfort or harm the study subjects in a given time. Therefore, reference was made to the general health law in its second title “On the ethical aspects of research on human beings”, the following articles: Article 13, 17, 18, 20, 21, which addresses study subjects as beings who must prevail in the criteria of respect, dignity, and protection of their rights and well-being; this was considered a risk-free study, coupled with the fact that informed consent was applied, and explained in a clear and precise manner. Lastly and in compliance with the General Law of Protection of Personal Data in Possession of Obligated Subjects and the Law of Protection of Personal Data Possession of Obligated Subjects for the State of Quintana Roo, the data collected was used solely for research purposes, with the research team assuming the legal and security measures to protect the personal data of the participants. 

The results were analyzed with the Statistical Package for Social Sciences (SPSS) version 25 for Windows 2010. Absolute frequencies, proportions, and percentages were used. A distribution analysis of the continuous variables was carried out with the Kolmogorov Smirnov test, categorizing the variables as non-parametric. Moreover, given the normality of the data to determine the difference in means by groups, the Mann-Whitney U test was applied. A structural equation model was tested using EQS v6.1 statistical software. The statistical indicator was the chi-square; if this relationship results with a significance level of *p* > 0.05, it was considered that the model has adequate statistical fit. Considering that (2 is usually susceptible to sample number, the relative (2 was used, which is calculated by dividing the adjusted χ2 index by the degrees of freedom. If this value is < 5, it was considered a good statistical fit. Furthermore, given that given that statistical indicators tend to be quite sensitive to sample size, The Comparative Fit Index (CFI), Bentler-Bonett Normed Fit (BBNFI) and Non-normed Fit (BBNNFI) were included, along with the Root Mean Square Error of Approximation (RMSEA).

The data collection process began with the request permissions in six public centers caring for the elderly; after that, the participants were selected randomly, applying a physical informed consent with the help of a research aide. Thereafter, and the study proceed to apply the aforementioned scales through a digital document. The dissemination of the results will be carried out after its publication through a community forum where society, civil associations, and universities will be in attendance. 

## Results

The study had 253 elderly participants with a mean age of 68 ± 7.5 years (95% CI= 67.1-68.9), with a mean of years living with the chronic disease of 10.14 years ± 10.5 years (95% CI= 8.8-11.4). Regarding the other demographic characteristics, there was prevalence of women (n = 152, 60.1%), primary or lower level of schooling (n= 130, 51.4%), with perceived medium economic level (n = 163, 64.4%), not caring for another person (n = 208, 82.2%), and having chronic diseases (n = 189, 78.3%), as seen in Table 1.

**Table 1:** Characterization of the 253 elderly participants

Variable	Category	n	%
Sex	Male	101	39.9
Sex	Female	152	60.1
Level of studies	Did not study	50	19.8
Level of studies	Primary	80	31.6
Level of studies	Secondary	42	16.6
Level of studies	High school	16	6.3
Level of studies	Technical career	24	9.5
Level of studies	Undergraduate	27	10.7
Level of studies	Specialization	5	2.0
Level of studies	Masterâ€™s	9	9.5
Economic level	Low	81	32.0
Economic level	Medium	163	64.4
Economic level	High	9	3.6
Caring for another person	Yes	45	17.5
Caring for another person	No	208	82.2
Suffers chronic disease	Yes	189	78.3
Suffers chronic disease	No	55	21.7
Prevalence of chronic diseases	Diabetes	79	41.7
Prevalence of chronic diseases	Hypertension	55	29.1
Prevalence of chronic diseases	Arthritis	16	8.4
Prevalence of chronic diseases	Asthma	14	7.4
Prevalence of chronic diseases	Cardiopathy	13	6.8
Prevalence of chronic diseases	Cancer	1	0.5
Prevalence of chronic diseases	Parkinsonâ€™s	1	0.5
Prevalence of chronic diseases	Other	10	5.6

Less alteration was found in the variables of self-distancing, self-transcendence, family functionality, and self-care agency with respect to that presented in the validation of the instruments (Table 2).

**Table 2:** Description of the scores of the variables studied in 253 elderly participants

Variable	M	SD	Max Val	Min Val	95% CI
Self-distancing	27.7	7.3	48	9	26.8-28.6
Self-transcendence	47.1	0.4	60	30	47.9-46.3
Family functionality	7.3	0.1	10	0	7.7-6.9
Self-care agency	86.8	988	120	36	88.8-84.9

Note: *M* = Mean, *SD* = Standard deviation, *Max Val* = Maximum value, *Min Val* = Minimum value, *95% CI* = Confidence Interval of 95% from the mean

Table 3 displays difference of means in the scores of self-distancing, self-transcendence, and selfcare, with the first variable being greater in the group without chronic diseases, while self-transcendence and selfcare were higher in the group with chronic diseases. 

**Table 3:** Difference of means of the variables studied according to the condition of having or not having a chronic disease

Variable	With chronic disease (*n* = 189)		Without chronic disease (*n* = 55)			*U*		***p -* value**
	*M*	*SD*	*M*	*SD*
Self-distancing	27.1	7.0	29.7	7.8	4.449.5	0.038
Self-transcendence	47.0	6.2	49.4	6.3	4177.0	0.008
Family functionality	7.3	8	7.5	2.8	5029.0	0.377
Self-care agency	87.1	13.8	91.6	12.7	4365.5	0.024

Note: *M* = Mean, *SD*= Standard deviation, *U* = Mann-Whitney U test, *p* = probability

The following structural model shows the effect of factors, like self-distancing (λ = 0.36), self-transcendence (λ = 0.50), and family functionality (λ = 0.54) on the self-care agency, demonstrating that these three constructs covaried with each other in positive and significant manner (*p* < 0.05). The factor loadings were high and significant (*p* < 0.05) indicating convergent construct validity for all the factors. Cronbach’s alpha for the entire set of indicators of self-transcendence, self-distancing, family functionality, and self-care agency resulted acceptable (α = 0.87). The goodness-of-fit indicators included a non-significant chi-squared (χ^2^ = 234.12 [145 *g.l.*], *p* = 0.123) and practical goodness-of-fit indicators close to 1 (*BBNFI = 0.95, BBNNFI = 0.95, CFI = 0.97*), added to the fact that the RMSEA value was 0.05. Self-distancing, self-transcendence, and family functionality explained 37% of the total variance explained of the factor of self-care agency, which is why all these values suggest that the theoretical model is backed by the data.

**Figure 1:**
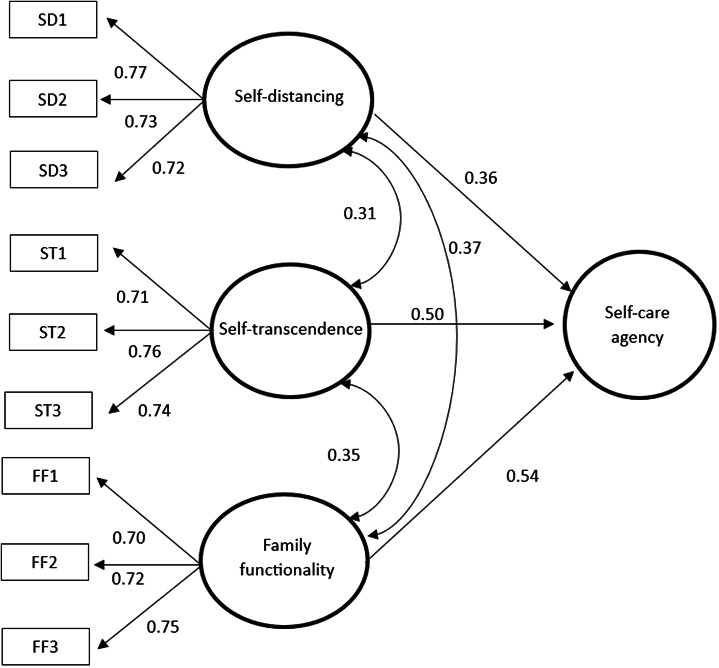
Structural model of the effect of self-distancing, self-transcendence, and family functionality on self-care agency

## Discussion

Social inequalities in the elderly have been affected in diverse areas, with health being one of them; this is different due to the biological, psychological, and social factors that determine functioning and the risk of falling ill, and it is unequal because norms and social values assign differentiated settings and roles to this age group, conditioning their life experiences, especially their health.^15^^,^^16^This research addressed factors that modify the self-care agency in the elderly, finding slightly high self-distancing, self-transcendence, and family functionality, confirming that conducted by researchers in the United States of America and Poland, which suggest that self-distancing is a differentiating factor for the strategies used by the elderly in their autobiographical reflection. When based on self-distancing strategies, the autobiographical reflection correlates with a higher sense of purpose in life and a higher level of self-care agency.^17^^,^^18^ Likewise, in Peru, researchers found high significance between the degree of family support and selfcare in the elderly because the relative or caregiver impacts on the support of the elderly, becoming an important part in caring for their disease.^19^^,^^20^

On the other hand, other studies carried out in Mexico by Guerrero *et al*.,^21^ as in Peru by Peralta *et al*.,^22^ highlight that self-transcendence generates grand benefits in all the spheres of the individual, such as at emotional, functional, social, spiritual and family level; for this reason, self-transcendence is considered valuable and inherent to humans to confront difficult situations in life, helping them to overcome and adapt adequately to the different stages of their existence. Thus, when the elderly have chronic diseases, they have greater self-distancing, while the elderly without pathologies have greater self-transcendence and better family functionality. This demonstrates that the fact of suffering a chronic disease modifies the elderly individual’s behavior, added to the mental health and the family function are also altered, with the disease factor being that which modifies the noological resources in this age group.^8^^,^^23^^-^^25^

Lastly, the study found that self-distancing, self-transcendence, and family functionality predict self-care agency, this is justified given that the elderly are essentially beings; within this it leads them to identify within themselves, and makes them in some way enter into a disturbing search for meaning to their own existence and essence, leading to self-reflection that permits identifying the positive and negative aspects that influence upon their health, which triggers maintenance or improves self-care.^26^^-^^28^

The importance of these results for the nursing practice lies in the possibility of developing educational interventions that enhance self-transcendence, self-distancing, and family functionality in the elderly. Said interventions may be designed to foster autobiographical reflection, family support, and emotional and social adaptation, thus, contributing to improve the self-care agency of this vulnerable population.^29^Nevertheless, these advances have significant challenges for the nursing profession, given that continuous and specific training is required in logotherapy that includes self-transcendence and self-distancing, as well as in communication skills and emotional support to effectively implement these interventions. Similarly, it is necessary to address the structural and social barriers that limit access by the elderly to health and community support resources.^30^Based on the foregoing, this study provides a solid base to improve knowledge and the nursing practice in relation to the self-care agency of the elderly from a behavioral perspective with participation by the family and nursing professionals, given that a key role is played in the promotion of health and wellbeing in this population, facing challenges with a holistic and emphatic approach. Among the limitations of the study was the lack of in-depth research on the topic in this age group, coupled with the fact that the size of the population is not updated at the state level, which could be a factor that modifies the sample size. Also, lack of government support was found to enter other public health care centers for the elderly to have a more random sample. 

This study concludes that self-transcendence, self-distancing, and family functionality explain 37% of selfcare in the elderly. In this sense, it is crucial for nursing professionals to implement health educational interventions focused on these behavioral factors and the family, given that by doing so, the self-care agency may be promoted and improved in this vulnerable population, which in turn will contribute to preventing the loss of functionality and to having greater independence in the elderly. 

These interventions must include strategies to strengthen self-transcendence and self-distancing, as well as to foster a functional and supportive family environment. Application of the results from this research in the nursing practice can result in a more holistic and effective approach in caring for the elderly, guaranteeing continuous and personalized monitoring of their health and wellbeing.
